# 

**Published:** 1879-01

**Authors:** 


### CONTENTS.

PAGE.

**CONTRIBUTIONS.**

Honor Bright, Ben. H. Pratt........87
The Issue as a Counter-irritant. P. H. Hayes, M. D..90
No Profession Exempt. Ausburn Towner........92
The Yellow Fever Question........93

**MEDICAL ITEMS AND NEWS.**

Creosote Wine—Ivy Poisoning—Hepatic Disease—Milk and Quinine—Intermittent Fever—Tobacco Sickness—Epilepsy—Inflammation of Bladder—Strychnia Poisoning—Sciatica—Nervous Sedative—Eczema—Pancoast's Styptic—Intestinal Obstruction—Opium Habit—Antiseptic Balsam—Vanquelin's Anti-asthmatic Cigarettes—Opium Poisoning—Hypodermic Solution of Morphia and Atropia—Epilepsy—Hiccough—Constipation Hæmoptysis—Chronic Cystitis—Sea Sickness—Glandular Sore Throat—Antidote to Strychnia—Whooping Cough — Membranous Vaganitis—Poultices—Salicylic Acid in Acute Rheumatism—Dysentery—The Hair—Salt Milk for Children—Eczema Rubrum—Chronic Alcoholism—Cholera—Tape Worm—Typhoid Fever—Cardiac Affection—Asthma—Dressing for Wounds—Epilepsy—Dysentery—Rheumatism—Cleansing Bladder—Alcoholism—Elastic Pencil of Caustic—Cement for Sealing Bottles........94 to 102

**OUR CORRESPONDENCE.**

Letter from Dr. Killpatrick........103

" " Dr. Dudley........104

Other Letters........103 to 104

PAGE.

**HOUSEHOLD HINTS AND RECIPES.**

Cheap Paint—Bright Scarlet Ink—Cement for Glass—To Remove Wool from Sheepskins—Hair Curling Liquid—Mushroom Catsup—Starch Gloss—Red Ink—Patent Leather Varnish—Saving Noise in a Sick Room—Liquid Blacking—Diet—Indelible Ink—Improved Black Writing Ink—Cement for Mending Rubber — Etching, Engraving or Writing upon Glass by Electricity—Leather Varnish—Indelible Ink—Cough Syrup—Cement for Aquaria—Hygene of Beds—To Restore Faded Writing—Damp Rooms—Coffee and Milk in Canine Distemper—Ebony Stain for Wood—Kerosene Liniment — New Liquid Glue — Mouth Washes........105 to 107

**EDITORIAL.**

Diphtheria........108

Ovarian Tumors........110

Christmas........111

**EDITORIAL CHIT-CHAT.**

Premiums—Relatives of Distinguished Men—Always Against the Woman — City Subscription List—Not Exempt—The Elmira Prison—Life Insurance Companies—Park Church Choir—Aquæ Gloria—Subscription Blank — Marriage of Old Folks—How Many Apples?—That History—Economy—Prison Camp—A Plucky Little Woman—Laughing Gas — Cooking Club— Fritz at School Stewart's Body........112 to 113

